# The feasibility and acceptability of integrating hepatitis C and HIV diagnostic testing on centralized molecular laboratory platforms in Myanmar

**DOI:** 10.1371/journal.pone.0282585

**Published:** 2023-05-17

**Authors:** Thandar Su Naing, Win Win Yee, Hlaing Thazin Aung, Khin Yi Oo, Khin San Tint, Caroline E. Boeke, Yuhui Chan, Magdalena Witschi, Nwe Nwe, Jessica Markby, Sonjelle Shilton, Khin Sanda Aung, Htun Nyunt Oo, Moe Myat Aye, Tin Tin Htay

**Affiliations:** 1 Clinton Health Access Initiative, Yangon, Myanmar; 2 National Health Laboratory, Department of Medical Services, Ministry of Health, Yangon, Myanmar; 3 Foundation for Innovative New Diagnostics, Geneva, Switzerland; 4 National Hepatitis Control Program, Department of Public Health, Ministry of Health, Naypyidaw, Myanmar; 5 National AIDS Program, Department of Public Health, Ministry of Health, Naypyidaw, Myanmar; Medecins Sans Frontieres, SOUTH AFRICA

## Abstract

**Background:**

In Myanmar, 1.3 million people have been exposed to hepatitis C (HCV). However, public sector access to viral load (VL) testing for HCV diagnosis remains limited; ten near-point-of-care (POC) devices are available nationally. Myanmar’s National Health Laboratory (NHL) has surplus capacity on centralized molecular testing platforms used for HIV diagnostics, presenting an opportunity for integrating HCV testing to expand overall testing capacity. This pilot assessed the operational feasibility and acceptability of HCV/HIV integrated testing implemented with a comprehensive package of supportive interventions.

**Methods:**

HCV VL samples were collected prospectively from consenting participants at five treatment clinics and tested at Myanmar’s NHL (October 2019-February 2020) on the Abbott m2000. To optimize integration, laboratory human resources were bolstered, staff trainings were offered, and existing laboratory equipment was serviced/repaired as needed. Diagnostics data during the intervention period were compared against HIV diagnostics data in the seven months prior. We conducted three time and motion analyses at the laboratory and semi-structured interviews with laboratory staff to assess time needs and program acceptability.

**Results:**

715 HCV samples were processed during the intervention period with an average test processing time of 18 days (IQR: 8–28). Despite adding HCV testing, average monthly test volumes were 2,331 for HIV VL and 232 for early infant diagnosis (EID), comparable to the pre-intervention period. Processing times were 7 days for HIV VL and 17 days for EID, also comparable to the pre-intervention period. HCV test error rate was 4.3%. Platforms utilization increased from 18.4% to 24.6%. All staff interviewed were supportive of HCV and HIV diagnostics integration; suggestions were made for broader implementation and expansion.

**Conclusions:**

With a package of supportive interventions, integration of HCV and HIV diagnostics on a centralized platform was operationally feasible, did not adversely impact HIV testing, and was acceptable to laboratory staff. In Myanmar, integrated HCV VL diagnostic testing on centralized platforms may be an important addition to existing near-POC testing in expanding national testing capacity for HCV elimination.

## Introduction

Worldwide, 58 million people are estimated to be living with chronic hepatitis C virus (HCV) infection, and 1.5 million new infections occur every year. In 2019, HCV-associated causes accounted for 290,000 deaths [1]. Although awareness of HCV is growing, diagnosis remains low; an estimated 21% of people living with HCV have been diagnosed globally, with 7% having been diagnosed in South-East Asia [2]. Effective strategies are needed to increase access to both HCV serology and HCV viral load (VL) testing for diagnosis.

In Myanmar, an estimated 1.3 million people have been exposed to HCV, yet most individuals are unaware of their status [3]. In 2017, Myanmar’s National Hepatitis Control Program (NHCP) established a national public HCV program with a National Strategic Plan for HCV screening, diagnosis and treatment. Direct acting antiviral (DAA) treatment is now available free of charge in 12 government facilities; treatment is also rapidly becoming more affordable in the private sector. However, despite this increase in the availability of treatment, case finding and diagnosis remain low in Myanmar. As of September 2020, an estimated 250,507 individuals have been screened for HCV antibodies, 34,913 individuals have tested positive for HCV antibodies, 15,471 individuals received confirmatory VL, and 14,205 individuals were diagnosed with chronic HCV [4]. While a number of factors have contributed to gaps in screening and diagnosis, a pilot study on HCV cost modelling by Burnet Institute reported that laboratory capacity constraints limited access to HCV VL, both for diagnosis and sustained virologic response testing (SVR12) to confirm cure after treatment [5].

Myanmar’s NHCP has 10 GeneXpert platforms (Cepheid, Sunnyvale, California) for HCV testing, which are relatively low-throughput and considered near-point-of-care (POC), designed for use at peripheral facilities. With these platforms available at hospitals across 7 states and regions in a hub-and-spoke model (facilities without devices send samples to facilities with devices for testing), there is currently a national capacity of 28,800 HCV VL tests per year, far short for the estimated 1.3 million of people needing to be tested within the population. The World Health Organization’s (WHO) 2022 Updated Recommendations on Simplified Service Delivery and Diagnostics for Hepatitis C Infection recommends the use of near-POC platforms as an “alternative approach to laboratory-based HCV RNA NAT assays to diagnose HCV viraemic infection” [6]. Comparatively, platforms designed for use in centralized molecular diagnostic laboratories have higher throughput and can cost less per test compared to near-POC [7]. While these centralized platforms generally require higher set up costs, more expertise and laboratory infrastructure, and have longer test processing times than near-POC platforms, utilization of both types of platforms may be crucial to scale up diagnostics coverage nationally. In a setting like Myanmar with a limited number of near-POC platforms conducting HCV VL testing in the public sector, integrated testing on centralized platforms could be an important approach to expand the national testing capacity, in addition to continuing to use the existing network of near-POC devices. This approach could also improve patient care services for HIV/HCV co-infected patients by leveraging the existing platforms and sample referral network of the HIV program.

The WHO and The Global Fund to Fight AIDS, Tuberculosis and Malaria recommend integration of multi-disease testing on the same platforms to increase device utilization efficiency and decrease costs [8–10]. These cost savings may be reinvested into case finding and other diagnostic priorities to increase testing access for underfunded programs. In addition, programs may have increased negotiating power with suppliers because of increased volumes by using multiplex platforms across multiple diseases.

While evidence on the benefits of integrated testing exists for other disease programs, particularly tuberculosis (TB) and HIV [11, 12], there is limited evidence or guidance on HCV/HIV diagnostic integration, particularly in the context of a national program. In Nigeria, an operational pilot study indicated that integrated HIV/TB/HCV testing using near-POC was possible and may contribute to improved program outcomes [13]. In Georgia, a pilot study of HCV/TB integration on near-POC reported that integration was feasible and did not have detrimental impacts on TB diagnostics [14]. Evidence for the utility and feasibility of integration of testing using high throughput testing platforms, however, remains limited.

Myanmar’s National Health Laboratory operates centralized testing for HIV VL and early infant diagnosis (EID) on two Abbott m2000 platforms with substantial spare capacity (>50%, per pre-intervention data). TB is diagnosed through a different program. These platforms offer an opportunity to integrate HCV and HIV diagnostics programs to expand national HCV testing capacity beyond the existing network of near-POC devices. This pilot study sought to assess the feasibility and acceptability of integrated HCV VL testing with HIV VL and EID testing on a centralized platform in the National Health Laboratory in Myanmar, when implemented alongside a comprehensive package of support that included additional human resources (HR), laboratory flow optimization, and training. We assessed whether reasonable HCV VL test volumes and times were achieved without adversely impacting HIV test volumes and processing times. Laboratory staff were also interviewed for feedback on the program.

## Methods

### Study design

The study was designed as a process evaluation to assess an integrated testing pilot which occurred at Myanmar’s National Health Laboratory over a period of four months (October 2, 2019 to February 4, 2020). A package of interventions was implemented to integrate HCV testing on centralized platforms being used for HIV VL and HIV EID testing. Before implementing the pilot, a time and motion analysis was conducted to understand laboratory processes and identify interventions needed to enable the addition of HCV VL testing to ongoing HIV VL and EID testing. Two observational time and motion analyses were subsequently conducted during the intervention period to track the progress of diagnostic integration. Routinely collected laboratory data (sample volumes, turnaround times (TAT), and error rates) on HCV VL were assessed during the pilot. In addition, routinely collected laboratory data on HIV VL and EID testing were compared before and during the intervention period (pre-intervention period: March 1, 2019 to September 30, 2019); intervention period: October 2, 2019 to February 4, 2020). Finally, at the end of the intervention period, semi-structured interviews were conducted with all seven laboratory staff involved in HCV, HIV VL, and EID testing to assess program acceptability.

### Study setting and participants

HCV VL samples were collected prospectively by onsite laboratory staff at all five public sector HCV treatment clinics in Yangon (Yangon Specialty Hospital, Waibargi Specialist Hospital, North Okkalapa General and Teaching Hospital, Thaketa Specialist Hospital, and Mingalardon Specialist Hospital) and were shipped to Myanmar’s National Health Laboratory, where the laboratory pilot program took place. Individuals were eligible to participate in this study if they were receiving HCV VL testing after a positive anti-HCV antibody test or SVR12 testing to confirm cure and they provided written informed consent to conduct testing on the centralized platform (for the study) in addition to their primary HCV VL test for routine clinical care which was conducted on a near-POC platform. Other eligibility criteria included age ≥ 18 years and sufficient plasma sample volume collected to perform HCV VL testing on both the near-POC platform and centralized platform.

#### Sample collection, transportation, and processing

Samples were collected as part of routine blood sample collection by laboratory staff within each clinic. In addition to the 6ml of venous blood collected for testing on a near-POC platform as per the standard of care, an additional 3ml of venous blood was collected for testing on the centralized platform. Healthcare workers (HCWs) followed standard procedures for sample collection and referral per national guidelines [15], requiring plasma separation to be conducted at treatment sites prior to sample referral.

Samples were transported to the National Health Laboratory in a cold chain by hospital staff using public transport, alongside the relevant paper-based forms (i.e., national diagnostics requisition forms and informed consent forms).

At the National Health Laboratory, samples were tested on a near-POC platform (GeneXpert, Cepheid) for routine clinical care. The remaining plasma volume (2ml) was transferred to the HIV Molecular Section where the integrated testing pilot took place. Aliquoted samples were stored at -20°C until testing (minimal time to test a batch of 24 samples for a centralized platform). Integrated testing of HIV VL, EID samples, and HCV VL was conducted in accordance with the manufacturer’s instructions by trained personnel. Pilot samples collected were used for HCV VL testing only. Test results from the centralized platform (m2000) device were not returned to patients. The National Health Laboratory takes part in the External Quality Assessment Scheme (EQAS) provided by Centers for Disease Control and Prevention (CDC) for HIV VL testing and Australia’s National Reference Laboratory (NRL) for HCV VL testing.

### Baseline assessment at the National Health Laboratory

As part of baseline assessments, the first time and motion analysis was conducted over two consecutive full workdays in May 2019. A time and motion analysis is used to understand and determine whether existing lab hours, personnel and infrastructure are sufficient to handle the HIV VL, EID and HCV testing. Two study team members observed staff in the HIV section of the laboratory’s virology department while staff members performed routine molecular testing activities. Data such as the total number of platform runs per day, number and type of personnel present, and hands-on time (defined as time spent on all activities related to HIV/EID/HCV molecular testing, including sample preparation, extraction and amplification, laboratory maintenance, results preparation and clerical work) were recorded.

### Description of intervention package

A package of interventions to support the integration of diagnostic platforms was defined based on discussions with senior staff from the National Health Laboratory and the baseline assessment findings. Interventions included additional HR support, including one medical technologist and one data assistant (clerical work was shifted to a newly hired data assistant, freeing up medical technologists’ time); adaptations to laboratory procedures; introducing automated bar codes to aid with data recording and reporting on LAB ACCEX, an electronic laboratory information management system used nationally; repairs and upgrades to laboratory equipment; training for all staff engaged in HCV VL testing on Abbott m2000; and refresher training on HIV VL and EID testing. Negotiations with the supplier (Q Bioscience/Abbott, Abbott Park, Illinois) enabled some of the interventions including refresher trainings and equipment maintenance and repair. Several of the interventions included are in line with WHO recommendations on HIV and TB diagnostics integration [9].

S1 Table contains information on the systems in place prior to this integration pilot and the components included for the integration based on the findings from the baseline assessment. With the additional HR support and other interventions, during the pilot a maximum of three runs per day (96 tests each, including controls) was possible on the testing platform.

### Intervention follow-up assessments at the National Health Laboratory

Two additional observational time and motion analyses, each consisting of two full workdays were conducted in December 2019 and January 2020 to assess the uptake of interventions recommended from the baseline assessment, evaluate the impact of interventions made, and allow pilot personnel to better understand the process and flow required for successful integrated VL testing.

### Interviews with laboratory staff

After the conclusion of the pilot, semi-structured individual key informant interviews were conducted with all seven laboratory staff involved in HCV, HIV VL, and EID testing to assess the acceptability of integrated testing and identify operational challenges. Staff members included two microbiologists (providing oversight at the laboratory), four medical technologists, and one data assistant. Their length of service at the National Health Laboratory ranged from six months to 26 months. The interviews were conducted verbally and responses transcribed to paper by an external consultant after obtaining written informed consent from each participant. Staff names were not collected with the surveys and all findings were summarized and reported anonymously to maintain confidentiality.

### Data collection and analysis

LAB ACCEX was used to extract the information required from the pilot period on HCV VL, HIV VL, and EID testing data, including patient demographics, testing volumes, device utilization, error rates, and testing TAT from sample receipt at the laboratory to result dispatch. Data from the HIV VL and EID tests performed during the four months of the intervention period were compared with data from the seven months before the intervention. Laboratory sample identification numbers were reviewed during data collection to link individual samples across the near-POC and centralized platforms on LAB ACCEX and for data cleaning purposes. No personal identifying information was collected. Routine site visits to the laboratory and five participating HCV treatment sites were conducted for monitoring and source data verification.

For the analysis, proportions were calculated for binary data, means (standard deviation) for normally distributed continuous data, and medians (interquartile ranges, IQR) for skewed continuous data. Monthly test volumes were reported as mean (range). StataSE was used for data analysis. For quality assurance purposes, the concordance was assessed between HCV VL results obtained from the centralized platforms and near-POC results. Maximum platform capacity was estimated based on the number of runs possible within laboratory operation hours (approximately 09:00 to 17:00). The average hands-on time recorded during the time and motion analyses was calculated before and during the intervention.

Staff interviews were recorded, and field note transcripts were prepared verbatim. Manual inductive coding and thematic analysis were done. The key themes were not limited to just the anticipated themes but also covered all the additional information raised by the participants.

## Ethical approval

This study was approved by the Institutional Review Board at the Department of Medical Research, Ministry of Health and Sports in Myanmar (Ethics/DMR/2019/102), National Hepatitis Control Program, National Health Laboratory, National AIDS Program, and Advarra Institutional Review Board in the US (Pro00037911).

## Results

### Patient characteristics and HCV test outcomes

During the intervention period, 715 patient samples were tested for HCV. The median patient age was 49 years (interquartile range: 39–58). Out of all samples collected, 52.7% were used for diagnosis of viremia whereas 44.9% were used for SVR12. Over 99% of HCV test results were concordant across the testing platforms (centralized and near-POC platforms). Table 1 summarizes the HCV samples by demographic characteristics and test outcomes.

**Table 1:** Description of HCV pilot sample by demographic characteristics and test outcomes.

Characteristics	N	%
**Sex (N = 715)**		
Female	372	52.0%
Male	343	48.0%
**Age (N = 714)** ^**1**^		
<20	8	1.1%
20 - <30	63	8.8%
30 - <40	118	16.5%
40 - <50	183	25.6%
50 - <60	202	28.3%
60 - <70	101	14.1%
70+	39	5.5%
**District (N = 715)**		
East Yangon	348	48.7%
North Yangon	20	2.8%
South Yangon	347	48.5%
**Test Reason (N = 715)**		
Confirmation of viraemia	377	52.7%
SVR12	321	44.9%
Others^2^	17	2.4%
**Outcomes for tests confirming viraemia (N = 377)**		
Below Limit of Detection	2	0.5%
Undetectable	46	12.2%
Detectable	329	87.3%
**Outcomes for SVR12 tests (N = 321)**		
Below Limit of Detection	5	1.6%
Undetectable	294	91.6%
Detectable	22	6.9%
**Outcomes for tests conducted for other reasons (N = 17)**		
Below Limit of Detection	0	0.0%
Undetectable	12	70.6%
Detectable	5	29.4%

^1^One patient was missing age information

^2^Any follow up or repeated testing of VL after SVR 12

### HCV test volumes

Table 2 shows the HCV sample testing information during the intervention period. Due to error results, 32 (4.3%) HCV samples were re-tested, generating a total of 747 HCV tests that were analyzed during the study (average: 187 tests per month; range: 45–245). A single batch of samples generated 30 of the 32 errors on two initial run cycles, due to a lack of familiarity among staff with the calibration process of the new reagents and protocol.

**Table 2:** HCV, HIV VL and EID testing during the pre–intervention and intervention periods*.

Indicator	HCV	HIV VL		EID	
	Intervention	Pre-intervention	Intervention	Pre-intervention	Intervention
**Total number of patients tested**	715	12,675	9,259	1556	916
**Number of tests re-processed due to errors**	32	110	65	18	13
**Error rate**	4.3%	0.9%	0.7%	1.2%	1.4%
**Monthly average test volumes**	187 (Range: 45–245)	1,826 (Range: 1389–2159)	2,331 (Range: 2028–2479)	225 (Range: 3–527)	232 (Range: 165–350)
**TAT between sample receipt and results printing (Days)**	18 (IQR:8–28)	9 (IQR:6–12)	7 (IQR:6–9)	26 (IQR:19–54)	17 (IQR:14–21)

*TATs for error results could not be calculated given the structure of the data. The range of monthly test volume values only included data for errors entered into LAB ACCEX.

### HCV test processing times

The median turnaround time between sample receipt at the laboratory and results printing for HCV tests was 18 days (IQR: 8–28). This was broken down to 1) 13 days (IQR: 7–22) between sample receipt and test processing, 2) 3 days (IQR: 1–10) between test processing and results printing. TAT decreased over time to 7 days (IQR: 6–13) by the final month of the pilot.

### HIV test data comparisons

#### HIV test volumes

HIV test data during the pre-intervention and intervention periods are shown in Table 2. The average monthly HIV VL test volumes increased by 28% before and during the intervention periods from 1,826 (range: 1389–2159) to 2,331 (range: 2,028–2,479) tests, while average monthly EID volumes remained similar (pre-intervention: 225 tests; range: 3–527; intervention period: 232, range: 165–350). The error rate remained similar for HIV VL (0.9% versus 0.7%, respectively) and EID (1.2% versus 1.4%, respectively).

#### HIV test processing times

Median TAT for HIV VL tests was 9 days (IQR: 6–12) before the intervention compared to 7 days (IQR: 6–9) during the intervention period. EID test processing time was not adversely impacted during the pilot; it was 26 days (IQR: 19–54) before the intervention period and 17 days (IQR: 14–21) during the intervention period.

### Platform utilization and downtime

With a maximum capacity of three runs of approximately 93 tests per day (excluding controls) on each of the m2000 platforms, the overall laboratory monthly testing capacity/utilization rate was 11,160 tests. The utilization rate across both platforms was 18.4% during the pre-intervention period (HIV VL and EID only) and 24.6% during the intervention period (HIV VL, EID and HCV VL).

Platform downtime was 0.7 weekdays/month pre-intervention versus 1.25 weekdays/month in the intervention period. The results from the time and motion analysis showed that medical technologists’ hands-on time (defined as time spent on molecular-testing-related activities on the centralized platforms) processing test samples decreased from 13.9 minutes pre-intervention to 5.3 minutes during the intervention.

### Qualitative findings

All staff interviewed were supportive of the integration of HCV VL testing at the laboratory and pointed out that integration was useful, citing its potential to expand testing and achieve cost savings. There was a high level of trust in the quality of the HCV test results generated on the centralized platforms, with staff attributing the platform’s ability to identify errors as a key factor. However, laboratory staff identified challenges with increased workloads. Four out of seven respondents reported facing issues during the pilot including machine downtime, and fear of cross-contamination between HIV and HCV samples. Four staff members reported feeling overwhelmed, particularly at times when there were high HIV test volumes to process. However, all staff who provided insights into workload management noted that the staffing levels during the pilot were adequate to manage the increase in workload. When asked about future scale-up of integrated HCV and HIV diagnostics, staff interviewed anticipated that colleagues would likely have concerns given pre-conceived notions of vertical programs having their own single-disease-use platforms. To address this potential concern, they made several recommendations during the interviews, including ensuring sufficient HR to take on more tests, with clear job descriptions and scopes of work from the respective disease programs for which integration would take place. Suggestions also included adapting the content of the trainings provided to account for the experience of attendees (such as focusing on practical sessions for more experienced attendees), a dedicated separate space for HCV sample storage within the storage room, a separate computer for HCV data entry, and strong maintenance support for laboratory equipment.

## Discussion

With laboratory workflow adjustments, introduction of an automated data system, provision of training, and staff support, integrated testing was achieved during this pilot without negatively impacting HIV test volumes or processing times. HCV VL tests were processed with a median time of 18 days from sample receipt to result printing. Laboratory workers were supportive of this integration program as implemented. However, it is important to emphasize that in this program, integration was successful when implemented with a package of supportive interventions including staffing changes.

Our findings complement positive findings from integrated testing strategies piloted in HCV, HIV, and TB programs in low- and middle-income countries (LMIC) such as Georgia, Nigeria, and Zimbabwe, although those studies employed near-POC platforms rather than laboratory-based platforms. A pilot in Georgia of HCV/TB diagnostics integration using near-POC found that integration was feasible, with no errors seen in results from either type of test during the program [14]. A pilot in Nigeria using near-POC for integrated HIV/TB/HCV testing demonstrated the feasibility and potential benefits of this strategy as well [13]. A feasibility pilot for HIV/TB integration using near-POC in rural Zimbabwe found that test integration on this platform was feasible, assessed using test volumes, turnaround times, and error rates, as well as linkage to care [12]. Finally, another pilot in Malawi and Zimbabwe of integrated HIV EID/HIV VL/TB testing on near-POC platforms reported that integration allowed for HIV diagnostics to be added to the platform without adversely impacting existing TB testing program TATs, error rates, or throughput [11].

The HCV VL test processing time of 18 days is a reasonable starting point for the general population given the long latency periods for adverse outcomes from HCV. Test processing times decreased to 7 days in the final month of this pilot due to gains in laboratory efficiency over time and as initial laboratory challenges with machine calibration and downtime were addressed. Machine downtime during the pilot, which contributed to longer TATs during some periods, was attributed to several factors, including the platform’s failure to interpret results, an extractor alignment error, and unstable calibration of the platform installed with the maxCycle protocol. The turnaround times seen in this pilot were comparable to pre-pilot near-POC HCV VL turnaround times in the national program according to a survey finding from sample transport mapping of treatment facilities in Yangon (7 days). and comparable to or lower than those reported for centralized testing in other disease areas and country contexts [16, 17]. A same-day test result through on-site near-POC testing would be preferable to maximize the timely initiation of treatment for all patients. However, HCV VL test processing times of less than one month are not expected to substantially compromise the continuity of clinical care or influence patient outcomes given the natural history of the disease. In the context of the capacity limitations on POC platforms nationally in Myanmar and the large demand for testing, the test processing times observed in this pilot are adequate and will be an important strategy to address this testing gap.

The initial reluctance at the National Health Laboratory for integrating laboratory testing of HIV and HCV, in part from a preconceived idea that the testing platform belonged to one vertical program, was overcome after the intervention package was implemented. 100% of all staff interviewed expressed positive feedback about integrated testing on the centralized platform and the efficiencies gained in the testing process by the clearer task division among relevant staff. For instance, the interventions implemented in the pilot contributed to a substantial reduction (>50%) in lab tech hands-on time. The task shifting of clerical work to a data assistant freed up the medical technologist’s time despite the increase in sample volume. In addition, the medical technologists’ role was restructured and clear job descriptions were developed to clarify roles and responsibilities. In this pilot, integration was feasible and acceptable to staff when implemented in conjunction with several key interventions. Interviews with laboratory staff at the conclusion of the pilot and observations made across the time and motion analyses affirmed the importance of these concurrent interventions in ensuring a successful integration program.

This study had several limitations. First, as the interventions were implemented as part of a package, it is not possible to isolate which factors played the biggest role in successful integration, or confirm that integration would be successful without the supportive interventions. The pre-intervention period was longer and occurred in different months of the year than the intervention period, which may have impacted the indicators examined. The pilot occurred at a single national health laboratory with dedicated HR and a technical support team and therefore results may not be generalizable to other settings with more limited resources. Finally, as samples were run on the centralized platforms as part of a research pilot in duplicate with samples utilized for clinical care, it is possible that test processing times could be different in the context of routine testing. Future research isolating and comparing individual interventions in the setting of routine care will help determine the most effective interventions to prioritize for integration programs and increase the generalizability of findings.

## Conclusion

The results from this pilot suggest that establishing integrated HCV/HIV testing at a large, centralized laboratory with excess platform capacity was possible in Myanmar without adversely impacting testing for existing programs. Integrated centralized testing may be an effective strategy in addition to near-POC to increase national testing capacity for HCV in Myanmar and other LMIC. The pros and cons of centralized integrated testing must be considered in the context of existing near-POC platforms, if any. For diagnostic testing to be integrated successfully, laboratory-specific decisions and investments are needed including workflow optimization, adequate HR support, additional training for staff, and ongoing oversight and quality assurance. Depending on the setting, integrated testing may require substantial investment in laboratory improvements and/or HR to realize its intended impact. Important next steps for countries scaling up both near-POC and centralized testing programs include defining models of differentiated service delivery to ensure that higher-risk groups and test types are prioritized for faster results. For instance, near-POC testing may be used to prioritize diagnosis, particularly for populations engaging in high-risk behaviors or at risk of loss to follow up, whereas SVR12 testing may use centralized testing as it is less time-sensitive. Ultimately, integrated testing may lead to overall cost sharing between disease programs and enable potential cost savings through pooled procurement of assays across diseases and sharing other implementation costs. Additional research is needed to estimate the program cost savings possible with integrated testing. In Myanmar and other LMIC, expanding access to laboratory testing through integrated testing and other strategies will be critical to achieve HCV elimination.

## Supporting information

S1 Table Components included in interventions to support the integration of diagnostic platforms. (DOCX)

S2 Table Pilot HCV testing dataset. (XLSX)

## References

[1] Fact Sheet: Hepatitis C. 2022 June 24 [cited 2022 August 23]. Available from: https://www.who.int/news-room/fact-sheets/detail/hepatitis-c.

[2] Global progress report on HIV, viral hepatitis and sexually transmitted infections, 2021. Geneva: World Health Organization; 2021 [cited 2022 August 23]. Available from: https://www.who.int/publications/i/item/9789240027077.

[3] WHO Myanmar newsletter special for World Hepatitis Day. Yangon, Myanmar 2019 [cited 2022 August 23]. Available from: https://www.who.int/docs/default-source/searo/myanmar/factsheet-viral-hepatitis.pdf?sfvrsn=9286597b_4

[4] National Hepatitis Control Program, Ministry of Health, Myanmar. 2020.

[5] Scott N, Win TM, Tidhar T, Htay H, Draper B, Aung PTZ, et al. Hepatitis C elimination in Myanmar: Modelling the impact, cost, cost-effectiveness and economic benefits. Lancet Reg Health West Pac. 2021 Mar 23;10:100129. doi: 10.1016/j.lanwpc.2021.100129 ; PMCID: PMC8315611. 34327345 PMC8315611

[6] Updated recommendations on simplified service delivery and diagnostics for hepatitis C infection. 2022 June 24 [cited 2022 August 23] Available from: https://www.who.int/publications/i/item/9789240052697

[7] HCV Market Intelligence Report and Preliminary HBV Market Insights. 2021 August 25 [cited 2022 August 23]. Available from: https://www.clintonhealthaccess.org/news/hepatitis-c-report-2021/

[8] Global Fund Support for Coinfections and Co-morbidities. The Global Fund to Fight AIDS, Tuberculosis, and Malaria; 2015. [cited 2022 August 23]. Available from: https://www.theglobalfund.org/media/4167/bm33_11-co-infectionsandco-morbidities_report_en.pdf

[9] Considerations for adoption and use of multidisease testing devices in integrated laboratory networks: information note. World Health Organization 2017 [cited 2022 August 23]. Available from: https://apps.who.int/iris/handle/10665/255693.

[10] Updated Recommendations on HIV Prevention, Infant Diagnosis, Antiretroviral Initiation and Monitoring. Geneva: World Health Organization; March 2021 [cited 2022 August 23]. Available from: https://www.who.int/publications/i/item/9789240022232 33822559

[11] Wang M, Boeke CE, Rioja MR, Maparo T, Banda C, Chavula C, et al. Feasibility and impact of near-point-of-care integrated TB/HIV testing in Malawi and Zimbabwe. AIDS. 2021. Epub 2021/07/27. doi: 10.1097/QAD.0000000000003031 . 34310372

[12] Ndlovu Z, Fajardo E, Mbofana E, Maparo T, Garone D, Metcalf C, et al. Multidisease testing for HIV and TB using the GeneXpert platform: A feasibility study in rural Zimbabwe. PLoS One. 2018;13(3):e0193577. Epub 2018/03/03. doi: 10.1371/journal.pone.0193577 ; PubMed Central PMCID: PMC5834185. 29499042 PMC5834185

[13] al. ACSJAOAe. GeneXpert Pilot for HCV Viral Load Testing at Dalhatu Araf Specialist Hospital (DASH), A Tertiary Healthcare Facility in a High Prevalence State in Nigeria. African Society for Laboratory Medicine (ASLM) 2018.

[14] Japaridze MM J.; Ruiz R.; Gabisonia I.; Adamia E.; Shilton S. Novel approach to near POC testing for HCV RNA; integration of HCV RNA testing into existing near POC machines used in National TB program, Georgia. International Journal of Infectious Diseases. 2020;101.

[15] National Simplified Treatment Guidelines of Viral Hepatitis C Infection. National Hepatitis Control Program MoHS, Myanmar, editor. 2019 [cited 2022 August 23]. Available from: https://www.aidsdatahub.org/resource/simplified-treatment-guidelines-hepatitis-c-infection-myanmar

[16] Thiha S, Shewade HD, Philip S, Aung TK, Kyaw NTT, Oo MM, et al. Early infant diagnosis of HIV in Myanmar: call for innovative interventions to improve uptake and reduce turnaround time. Glob Health Action. 2017;10(1):1319616. Epub 2017/06/03. doi: 10.1080/16549716.2017.1319616 ; PubMed Central PMCID: PMC5496058. 28574336 PMC5496058

[17] Boeke CE, Joseph J, Atem C, Banda C, Coulibaly KD, Doi N, et al. Evaluation of near point-of-care viral load implementation in public health facilities across seven countries in sub-Saharan Africa. J Int AIDS Soc. 2021;24(1):e25663. Epub 2021/01/18. doi: 10.1002/jia2.25663 ; PubMed Central PMCID: PMC7811577. 33455081 PMC7811577

