# Supplementary material for: The feasibility and acceptability of integrating hepatitis C and HIV diagnostic testing on centralized molecular laboratory platforms in Myanmar
Source: PLoS One. 2023 May 17;18(5):e0282585. doi: 10.1371/journal.pone.0282585 (PMC10191262; doi:10.1371/journal.pone.0282585)
Supplement: S1 Table — (DOCX) [file pone.0282585.s001.docx]

**S1 Table. Components included in interventions to support the integration of diagnostic platforms**

| **Component** | **Baseline status before the intervention was implemented** | **Intervention package** |
| --- | --- | --- |
| Training | - Not applicable | - A 2-day training was provided on HIV and HCV viral load testing using the centralized PCR platform |
| Human Resources | - A portion of 5 staff members’ time went towards HIV testing with no clear job description. Due to limited HR capacity at NHL, staff performed dual functions such as HIV serological and molecular testing and other non-HIV testing (e.g influenza, dengue etc)   The following staff contributed to testing   - - 2 full-time medical technologists focused on HIV VL and EID testing   - 1 microbiologist (supervising the testing for all diseases at the laboratory)   - 1 part-time medical technologist (also performing other diseases testing at the laboratory   - 1 part-time laboratory assistant (contributed to clerical tasks and aid medical technologist in sample processing as required) | - Existing roles were restructured and clear job descriptions were developed   Updated personnel and roles in the laboratory were as follow:   - - 1 full-time medical technologist focused on HIV and HCV integrated testing   - 1 full-time data assistant focused on clerical tasks   - 1 part-time microbiologist (supervising the testing for all diseases at the laboratory)   - 1 medical technologist (also perform other diseases testing at the laboratory)   Note that there was staff turnover and rotation between the training and the end of the pilot (3 staff members transitioned) |
| Work Flow | - Standardized shift of 09:30 – 16:30 for all - Manual labelling of samples - Medical technologist engaged in diverse scope of work which mainly included the clerical tasks | - Work hours for one medical technologist were increased by 30 minutes when necessary - Automated barcodes were used to label samples - Data management, serology testing, and other responsibilities handled by medical technologist were shifted to other staff in the virology unit - The same workstation with biological safety cabinet was used for sample preparation for HIV and HCV samples. Medical technologists performed and followed good laboratory practice and safety measures to reduce the risk of contamination |
| Technology | - 1 well-functioning and 1 mal-functioning automated extractor (m2000sp) - 2 automated amplifiers (m2000rt) | - Automated extractor was prepared - maxCycle software was installed to enable multi-disease testing on the same run |
